# Supplementary material for: Monitoring the impact of confinement on hyphal penetration and fungal behavior
Source: PLoS One. 2024 Dec 30;19(12):e0312855. doi: 10.1371/journal.pone.0312855 (PMC11684722; doi:10.1371/journal.pone.0312855)
Supplement: Attachment — Submitted filename: PLOS One Rebuttal Letter October 2024.docx [file pone.0312855.s001.docx]

Scott T. Retterer

Center for Nanophase Materials Sciences

Oak Ridge National Laboratory

Oak Ridge, TN 37831-6493

(865) 405-4066 | rettererst@ornl.gov

October 10, 2024

Dear PLOS One editors,

Enclosed is a revised version of the manuscript entitled *“Monitoring the impact of confinement on hyphal penetration and fungal behavior”*, authored by Yi-Syuan Guo, Julian Liber, Jennifer Morrell-Falvey, Gregory Bonito, and myself, Scott T. Retterer. We would like to resubmit this manuscript for further consideration as a research article in *PLOS One*. All authors have reviewed and approved the revised version of this manuscript.

The reviewer acknowledged the significance of the work noting the “great theoretical importance” of the work as well as the “great practical importance” of the study. However, the reviewer questioned whether the results shown were “statistically significant”. In the previous version of the manuscript we had, in fact, noted that a student’s two tailed t-test was used to gauge the statistical significance of the difference between the penetration rates measured in the 5-µm versus the 10-µm wide microchannels. With p-values well below 0.005, as reported in the manuscript, the t-test supports that the observed differences were statistically significant. While all this information was reported in the original submission, we acknowledge that the text could have been clearer, and that additional information about the number of experimental replicates performed and number of measurements used to calculate these p-values would have been beneficial to the reviewer. Changes to the text and appropriate tables were made to improve the clarity of the portions of the article that described those aspects of the study and the subsequent analysis of the results. We thank the reviewer for their efforts and feel that we have addressed their concerns.

If you require any additional information, please contact me at the above address.

Sincerely,

Scott T. Retterer, Ph.D.

Distinguished Staff Scientist

Center for Nanophase Materials Sciences

Oak Ridge National Laboratory
